# Current-induced SQUID behavior of superconducting Nb nano-rings

**DOI:** 10.1038/srep28320

**Published:** 2016-06-20

**Authors:** Omri J. Sharon, Avner Shaulov, Jorge Berger, Amos Sharoni, Yosef Yeshurun

**Affiliations:** 1 Department of Physics and Institute of Nano Technology, Bar-Ilan University, 5290002 Ramat-Gan, Israel; 2 Department of Physics and Optical Engineering, Ort Braude College, 21982 Karmiel, Israel

## Abstract

The critical temperature in a superconducting ring changes periodically with the magnetic flux threading it, giving rise to the well-known Little-Parks magnetoresistance oscillations. Periodic changes of the critical current in a superconducting quantum interference device (SQUID), consisting of two Josephson junctions in a ring, lead to a different type of magnetoresistance oscillations utilized in detecting extremely small changes in magnetic fields. Here we demonstrate current-induced switching between Little-Parks and SQUID magnetoresistance oscillations in a superconducting nano-ring without Josephson junctions. Our measurements in Nb nano-rings show that as the bias current increases, the parabolic Little-Parks magnetoresistance oscillations become sinusoidal and eventually transform into oscillations typical of a SQUID. We associate this phenomenon with the flux-induced non-uniformity of the order parameter along a superconducting nano-ring, arising from the superconducting leads (‘arms’) attached to it. Current enhanced phase slip rates at the points with minimal order parameter create effective Josephson junctions in the ring, switching it into a SQUID.

Small-size SQUIDs attract considerable interest for investigations of local magnetic signals, measuring, e.g., dynamics and pinning of single vortices^1^,^2^ and local superfluid density in superconductors^3^, ferromagnetic patches at the LaAlO_3_/SrTiO_3_ interface^4^, quantum magnetization reversal of ferromagnetic nanoparticles^5^ and single molecule magnets^6^. A few designs of small SQUIDs without Josephson junctions have been proposed, based on mesoscopic superconducting loops^7^, asymmetric superconducting rings^8^, inhomogeneous superconductors^9^, constrictions in the superconducting rim^10^, interrupted mesoscopic normal loop in contact with two superconducting electrodes^11^, and a combination of superconducting and metallic contact banks^12^. SQUIDs without Josephson junctions may offer advantages in simplicity of fabrication, and, under certain conditions, a steeper dependence of the measured quantities on the magnetic flux^8^. The present study may offer a different approach in designing a SQUID without Josephson junctions by switching a nano-ring with two arms into a SQUID using a large enough bias current. The potential application of such a SQUID will be discussed elsewhere. Here we focus on the fundamentally important observation of current-induced switching between Little-Parks and SQUID magnetoresistance oscillations in Nb nano-rings.

Niobium amorphous films, 40 nm thick, were deposited from a Nb target on silicon substrates via DC-magnetron sputtering. The films were patterned into square rings (side 340 nm, rim’s width 55 nm) with two arms (65 nm wide, 250 nm long) as shown in Fig. 1. For signal amplification, a string consisting of a serial connection of 260 such rings was measured. Details of the sputtering and patterning processes are described in the *Methods* Section. Measurements were performed near the superconducting transition temperature for bias currents between 10 nA and 10 μA, employing a commercial Physical Properties Measurements System (PPMS, Quantum-Design).

Figure 2 shows the temperature dependence of the resistance of a single ring with two arms measured at zero applied magnetic field. The curve corresponding to the lowest measuring current exhibits a sharp transition at T_c_ ~ 7.2 K with a width ≼ 0.1 K. As the current increases, the R(T) curves are shifted to lower temperatures, as expected. The right inset to Fig. 2 shows the temperature dependence of the sample resistance over an extended temperature range, between room temperature and 4 K, using measuring current of 10 nA.

Figure 3 shows typical magnetoresistance oscillations measured at T = 7.1 K, representing three different types of waveforms obtained for different measuring currents. At low currents (1 μA and below) classical Little-Parks oscillations^13^ are obtained, exhibiting parabolic shape with *upward* cusps at odd multiples of *Φ*_*0*_*/2*, and a field-period of ~170 Oe, corresponding to the area of a single ring (~1.2 · 10^−9^ cm^2^). For higher currents, in the range ~2–3 μA, the cusps disappear and the oscillations become sinusoidal. As the current further increases to 4 μA, the waveform drastically changes, exhibiting *downward* cusps at multiples of *Φ*_*0*_, typical of the magnetoresistance response of a SQUID biased with a current that is equal to its maximum supercurrent^14^. The data of Fig. 3 can thus be well interpreted as indicating a current induced switching of the ring into a SQUID. Similar results were obtained in a narrow temperature range between 7 and 7.15 K. However, the current required to obtain the SQUID response sharply increased with decreasing temperature, such that below 7 K it reached a level beyond the limit of our system. In addition, we performed measurements at constant currents varying the temperature between 6.5 and 7.2 K. These measurements showed that SQUID like magnetoresistance oscillations can also be obtained by increasing the temperature biasing the sample at a high current.

To better illustrate the current-induced switching between Little-Parks and SQUID magnetoresistance oscillations, we add in Fig. 3 guide to the eye solid curves through the data points, based on theoretical predictions for the various types of magnetoresistance oscillations, Δ*R*. The theoretical curves are superimposed on the measured monotonous background. The lowest curve (1 μA) in Fig. 3 describes typical Little-Parks parabolic oscillations (see, e.g. Figure 4.5 in ref. 15):


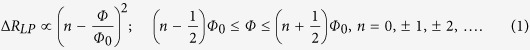


The intermediate curves (2 and 3 μA), correspond to the Little-Parks oscillations in a ring with two symmetric Josephson junctions (i.e., a SQUID), calculated on the basis of the J^2^ model^16^. According to this model, the magnetoresistance oscillations in a single superconducting loop follow the field dependence of the square of the screening current *I*_*s*_. Thus, the linear field dependence of *I*_*s*_ in a simple ring gives rise to parabolic oscillations. However, in a SQUID the screening current is sinusoidal, |I_s_| = I_c_ |sin(πΦ/Φ_0_)|, where I_c_ is the critical current of each of the Josephson junctions forming the SQUID^17^. Accordingly, the Little-Parks oscillations in a SQUID are expected to be sinusoidal:


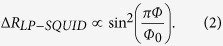


Note that in a conventional SQUID it is assumed that the rim width is larger than the superconducting penetration depth, λ, and, therefore, the Little-Parks effect is unobservable. However, in our case, as is virtually the case in all nano-rings, the rim width is smaller than λ. Thus, magnetoresistance oscillations due to Little-Parks effect, Eq. (2), are expected in such SQUIDs near T_c_.

The upper solid curve in Fig. 3, corresponding to 4 μA, is based on the formula for the average voltage across a SQUID in the dissipative regime,


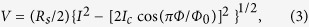


where *R*_*s*_ is the junction’s shunt resistance and *I*_*c*_ is the critical current of each of the Josephson junctions (see Eq. 6.48 in Ref. 15). Assuming that the external current *I* = 2*I*_*c*_, one obtains:


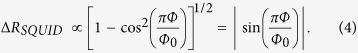


The agreement between the calculated curves and the data shown in Fig. 3 supports our scenario of current-induced switching of a superconducting nano-ring with two arms into a SQUID.

The data of Fig. 3 indicate that the crossover from a ring to a SQUID occurs for currents around 2 μA, where the parabolic Little-Parks oscillations transform to sinusoidal oscillations. As noted above, the bias current of ~4 μA corresponds to the SQUID response for external current that equals the maximum supercurrent of the SQUID, *I*_*m*_*(Φ* = *0)* = *2I*_*c*_. Indeed, the I-V curve measured at 7.1 K in zero field (see left inset to Fig. 2) shows an abrupt voltage increase around this current. It should be noted that the bias current of 4 μA includes the current (~2 μA) needed to transform the ring into a SQUID. Thus, the maximum supercurrent of the SQUID, *I*_*m*_(*0*), is approximately 2 μA. The dependence of *I*_*m*_ on *Φ* can be deduced from the data of Fig. 3, using Eq. (3). The results are shown in Fig. 4 together with the theoretical *I*_*m*_*(Φ)* in a conventional SQUID (solid line). The theoretical curve is superimposed on the bias current *I*_*0*_ required for the creation of our SQUID:


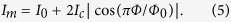


In deriving *I*_*m*_(*Φ*), the background was subtracted and the junction’s shunt resistance for a single SQUID was estimated as R_S_ = 2.3 Ω, corresponding to twice the measured resistance of the SQUID at *Φ*_0_/2. The oscillatory behavior of the critical current above a bias current of 2 μA seems to compare quite well with that of a real SQUID with two Josephson junctions (solid curve in Fig. 4). Note, however, that our current-induced SQUID does not show the plateau behavior of *I*_*m*_(*Φ*) around integer flux quanta as proposed in Refs 7 and 18.

The current induced switching of a superconducting nano-ring with two arms into a SQUID can be understood considering the non-uniform order parameter along the ring in such a structure when a magnetic field is applied^19^,^20^,^21^,^22^,^23^. Based on the Ginzburg-Landau equations, de-Gennes^19^ and Alexander^20^ showed that two minima of the order parameter are generated at equal distances from the connection points of the arms to the ring^20^,^21^. An intuitive way to understand this result is by starting with a ring with a single arm. As the arm is not affected by the magnetic flux, the order parameter along the ring has a maximum at the connection point and a minimum at the antipodal point. This minimum drops to zero at the onset of superconductivity when the flux becomes equal to a half flux quantum, *Φ* = *Φ*_*0*_*/2*. When two symmetrical arms are connected to a ring, the order parameter is maximum at the connection points and minimum at equal distances from these points^20^,^21^. Solving the nonlinear Ginzburg-Landau equations, Fink *et al.*^18^ predicted that the maximum supercurrent in a ring with two arms of size comparable to the coherence length, depends periodically on *Φ* in a way similar to a classical SQUID. Experimental confirmation of this prediction was demonstrated in Al mesoscopic rings using current-voltage measurements^7^. The previous studies^7^,^22^ emphasized the role of the geometrical parameters of the ring and did not consider the role of the bias current in inducing the SQUID behavior. Our magnetoresistance measurements indicate that the geometry alone is insufficient to produce a SQUID without the involvement of large enough bias current. This is evident from the classical parabolic Little-Parks oscillations observed at low bias current (lowest curve in Fig. 3). As explained above, formation of a SQUID at these low bias currents would result in sinusoidal rather than parabolic Little-Parks oscillations. Such sinusoidal oscillations are observed only when the bias current is increased to ~2 μA.

The role of the bias current in inducing a SQUID behavior can be associated with current-induced phase slips^24^. Currents above *I*_*m*_(*Φ*) generate a voltage drop across the SQUID. Due to the position dependence of the voltage, the phase of the order parameter changes at different rates in different places, leading to phase slips that are more effective at the points with weakest superconductivity in the circuit. Phase slips at these points further reduce the order parameter down to a level required for the creation of effective Josephson junctions. Note that the current passes asymmetrically through some of the loops sitting at the right and left sides of the array, see Fig. 1. However, as shown in refs 8 and 25, except for skewness the behavior of an asymmetric loop is similar to that of a symmetric loop.

For the creation of the Josephson junctions within the range of a single *Φ*_0_, the weak links must be limited to a length scale comparable to the coherence length, ξ(T). Furthermore, for the phase slips to be effective, the rim’s cross-section should be of order ξ^2^(T). These requirements are satisfied at temperatures close to T_c_ where ξ(T) = ξ_0_(1 − T/T_c_)^−1/2^ = 340 nm at T = 7.1 K, taking the zero temperature coherence length ξ_0_ = 37 nm^10^. Finally, we note that the possibility of coupling of the response of neighboring rings due to non-local effects is excluded because the distance between neighboring rings is an order of magnitude larger than ξ_0_. Non-locality between neighboring rings due to the magnetic field generated by the rings is also excluded, since close to T_c_ the screening currents are negligibly small.

In conclusion, we have demonstrated that a superconducting nano-ring with two arms can be switched into a SQUID by externally applied bias current. The SQUID behavior was demonstrated by the current induced transformation of the Little-Parks magnetoresistance oscillations from parabolic into sinusoidal oscillations and eventually into oscillations typical of a SQUID. The formation of a SQUID is attributed to the combined effects of current induced phase slips and non-uniform order parameter along the ring caused by the superconducting arms. We note that such superconducting structures comprising a ring with two arms are common in nano-fabrication in which the arms serve as leads to the ring. Such superconducting nano-structures may be utilized as field sensitive nano-devices without artificial Josephson junctions.

## Methods

Niobium thin films were deposited from a Nb target (99.95%, ACI Alloys) on silicon substrates with 1 μm of thermal silicon oxide via DC-magnetron sputtering. Sputtering was performed in 2 mTorr Ar pressure, at room temperature and a rate of 1.8 Å/s, to a total thickness of 40 nm. Subsequently, the Nb films were spin-coated with about 200 nm of Poly(methyl ethacrylate) (PMMA) 950 A4 resist (Microchem Corp.) and baked on a hot-plate at 180 °C for 120 seconds.

Masks were made using Electron-Beam Lithography by overexposing the PMMA and causing the PMMA polymers to be cross-linked as described in^26^,^27^. The Nb was etched with Reactive Ion Etching (RIE) using SF_6_. The full procedure is described in^28^. Electrical contacts were made by wire bonding of 25 um thick aluminum wires directly to the Nb.

## Additional Information

**How to cite this article**: Sharon, O. J. *et al.* Current-induced SQUID behavior of superconducting Nb nano-rings. *Sci. Rep.* **6**, 28320; doi: 10.1038/srep28320 (2016).

## Figures and Tables

**Figure 1:**
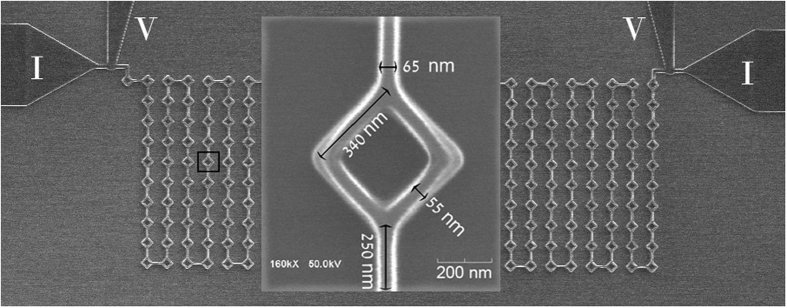
A Scanning Electron Microscope (SEM) image of a single ring with two arms. The background shows the serially connected rings with the current and voltage leads.

**Figure 2:**
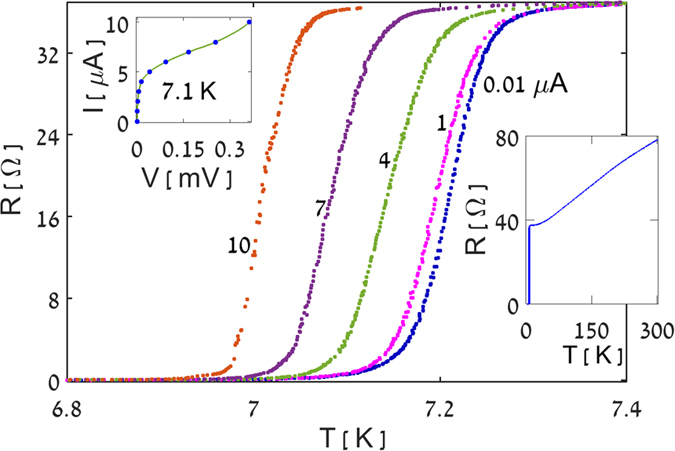
Resistance of a single ring with two arms versus temperature measured with different bias currents. Right inset: Temperature dependence of the resistance in an extended temperature range for I = 10 nA. Left inset: I-V curve measured at 7.1 K in zero field.

**Figure 3:**
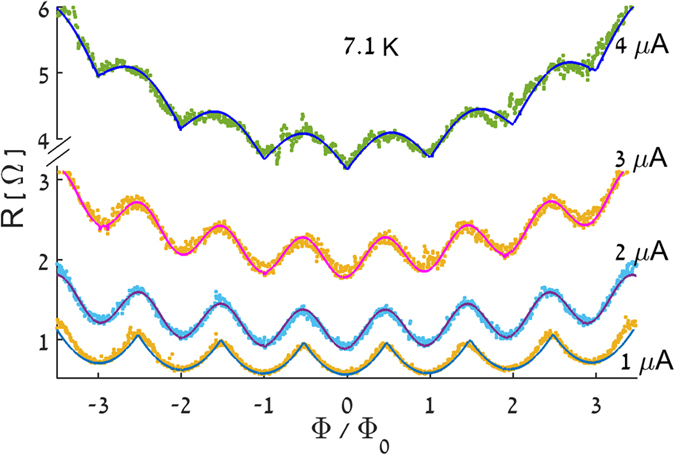
Magnetoresistance of a single ring for different bias currents. Measurements performed at T = 7.1 K with currents between 1 μA and 4 μA are described as a function of the magnetic flux, Φ, normalized to the quantum flux, Φ_0_, taking the ring area as 1.2 · 10^−9^ cm^2^. The guide to the eye solid curves through the data points describe classical Little-Parks parabolic oscillations (1 μA curve), Little-Parks sinusoidal oscillations in a SQUID (2 and 3 μA curves) and typical SQUID oscillations (4 μA curve), Eqs (1), (2) and (4), respectively, superimposed on monotonic backgrounds.

**Figure 4:**
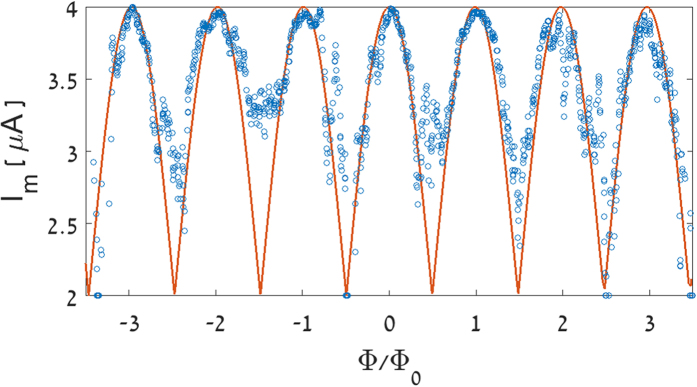
The maximum supercurrent *I*_*m*_ of the current-induced SQUID as a function of the normalized flux. The solid curve describes the theoretical *I*_*m*_*(Φ)* in a conventional SQUID superimposed on the bias current *I*_*0*_ required for the creation of the SQUID, Eq. (5). The junction’s shunt resistance R_S_ is taken as 2.3 Ω.
